# The Effects of Peanut Oligopeptides on Exercise-Induced Fatigue in Mice and Its Underlying Mechanism

**DOI:** 10.3390/nu15071743

**Published:** 2023-04-02

**Authors:** Rui Liu, Zhen Li, Xiao-Chen Yu, Jia-Ni Hu, Na Zhu, Xin-Ran Liu, Yun-Tao Hao, Jia-Wei Kang, Yong Li

**Affiliations:** Department of Nutrition and Food Hygiene, School of Public Health, Peking University, Beijing 100191, China

**Keywords:** peanut peptides, fatigue, antioxidant, energy metabolism

## Abstract

The aim of this study was to clarify the anti-fatigue effect of peanut oligopeptides (POPs) in mice and to investigate its possible underlying mechanism. A total of 150 male ICR mice were randomly assigned into five groups: control, whey protein (0.50 g/kg·bw), and three peanut peptide groups (0.25, 0.50, and 1.00 g/kg·bw). All the mice were treated with intra-gastric administration for 30 days. Following the intervention, a weight-loaded swimming test, blood lactate concentration, glycogen content, the activities of antioxidant factors and energy metabolism enzymes, and the function of mitochondria in the skeletal muscle were examined. The results show that POP intervention significantly prolonged the exhaustive swimming time, decreased blood lactate concentration levels, regulated the process of energy metabolism, and increased the level of antioxidant enzymes, muscle glycogen, and expressions of mtTFA and NRF-1 in the mitochondria of the gastrocnemius muscle. The results suggest that POPs produce an anti-fatigue effect in the animals, and they may exert this effect through the mechanism of improving the animals’ antioxidant capacity to reduce oxidative damage levels and regulating the process of energy metabolism.

## 1. Introduction

Fatigue refers to the subjective discomfort following prolonged or strenuous exercise, which is a reflection of the decrease in the level of physiological functions and the ability to maintain the functioning of the body [1]. Chronic fatigue states can cause immune deficiency, endocrine disorders, and cognitive impairment, which eventually develop into chronic fatigue syndrome (CFS) [2]. At present, in the United States, 2.3% of men suffer from CFS [3]. In a study, Shi J observed that 12% of secondary school students in China suffered from chronic fatigue [4]. Fatigue is mainly related to the stressful metabolic enhancement of the body caused by exercise and other related factors, such as a high-level of consumption of energy substances, the accumulation of metabolic substances in the body, oxidative stress damage, and protective central inhibition [2]. In addition, fatigue is also a complication of several diseases and is closely related to the patient’s recovery process; therefore, it is very important to investigate the mechanism of fatigue and explore effective anti-fatigue solutions in the literature. Researchers have determined that sensible nutritional measures can effectively promote the recovery of muscle function, enhance oxygen utilization, and delay the progression of fatigue in individuals [5,6,7]. However, numerous nutrients do not result in improved anti-fatigue effects on the body due to their single function and low absorption and efficiency rates. Therefore, it is of great importance for scientists to explore natural nutrient active substances with multiple physiological activities that can efficiently exert anti-fatigue effects.

In recent years, bioactive peptides have become a hot topic of research in the field of nutrition due to their easy absorption and multiple physiological roles [8,9]. Bioactive peptides have a faster absorption rate and higher absorption efficiency than proteins and can rapidly participate in energy metabolism, provide good nitrogen sources to the body, and participate in the oxidative energy supply by generating α-keto acids through the process of oxidative deamination [8,10,11]. It has been observed in the literature that ginseng, sea cucumber, and walnut oligopeptides, as well as tilapia collagen peptides, can decrease blood lactate levels in mice and produce anti-fatigue effects [5,6,7,12,13].

Peanuts are rich in oils, proteins, and a variety of bioactive substances, and their protein content accounts for approximately 24% to 36% of the total amount, which is a high-quality source for the extraction of bioactive peptides [14,15]. Peanut oligopeptides (POPs) are extracted from peanut proteins by using bienzymatic techniques [16]. POPs have better stability and solubility properties than peanut proteins and can participate in energy metabolism and provide a rapid source of nitrogen to the organism [17,18,19]. POPs are also rich in lysine, aspartic acid, and glutamic acid [20]. Lysine acts as an electron acceptor to neutralize free-radical electrons generated during the oxidation of unsaturated fatty acids, while aspartic and glutamic acids can chelate metal ions and inhibit the occurrence of oxidative stress [21]. POPs also present potential hypotensive activity, and peptide bonds consisting of hydrophobic amino acids are preferentially hydrolyzed during the enzymatic preparation of peanut peptides, resulting in a higher content of hydrophobic amino residues in peanut peptides, thus possessing better angiotensin-converting enzyme-inhibitory activity [18]. POPs have great potential for development in the field of functional foods and pharmaceuticals due to their good physiological activity. Studies on the anti-fatigue effects of POPs have rarely been reported in the literature and there remains a lack of in-depth research on this subject. Therefore, in this study, we use ICR male mice to investigate the anti-fatigue effects of POPs and explore the potential underlying mechanisms.

## 2. Materials and Methods

### 2.1. Materials and Reagents

Peanut oligopeptides (POPs) were extracted from peanut proteins using biological enzymatic technology, of which small-molecule peptides with a relative molecular mass less than 2000 Da accounted for more than 80% and were purchased from Wuhan Tallyho Biological Product Co., Ltd. (Wuhan, China). Lactate dehydrogenase (LDH) (A020-2-2), blood urea nitrogen (BUN) (C013-2-1), creatine kinase (CK) (A032-1-1), glucose (A154-2-1), superoxide dismutase (SOD) (A001-3-2), glutathione peroxidase (GSH-PX) (A005-1-2), malondialdehyde (MDA) (A003-1-2), liver/muscle glycogen (A043-1-1), pyruvate kinase (PK) (A076-1-1), malate dehydrogenase (MDH) (A021-1-1), succinate dehydrogenase (SDH) (A022-1-1), Ca^2+^-Mg^2+^-ATPase (A070-6-2), and Na^+^-K^+^-ATPase (A070-2-2) assay kits were purchased from the Nanjing Jiancheng Biotechnology Institute (Nanjing, China). The sodium fluoride was purchased from Nanhai Shuangfu Chemical Co., Ltd. (Foshan, China), and *p*-hydroxy biphenyl was purchased from Shandong Xihua Biotechnology Co., Ltd. (Zhouping, China). All other reagents used in the study were of analytical grade.

### 2.2. Animals and Experimental Design

One hundred and fifty healthy specific-pathogen-free (SPF)-grade ICR male mice, 6~8 weeks old with an initial weight of 28~32 g, were purchased from the Experimental Animal Center, School of Public Health, Peking University. The animals were housed in a barrier-level animal room in the Experimental Animal Center of Peking University Medical Department with a temperature range of 22 ± 2 °C, relative humidity of 50–60% RH, and day/night alternation time of 12 h:12 h and were fed and given water freely during the experimental period. The experiment was reviewed and approved by the Institutional Animal Care and Use Committee of Peking University, and all animals were treated according to the Principles of Laboratory Animal Care (NIH publication No. 85-23, revised 1985) and the guidelines of the Peking University Animal Research Committee (Laboratory animal production license No.: SCXK (Jing) 2016-0010; Laboratory animal use license No.: SYXK (Jing) 2016-0041).

**Figure 1:**
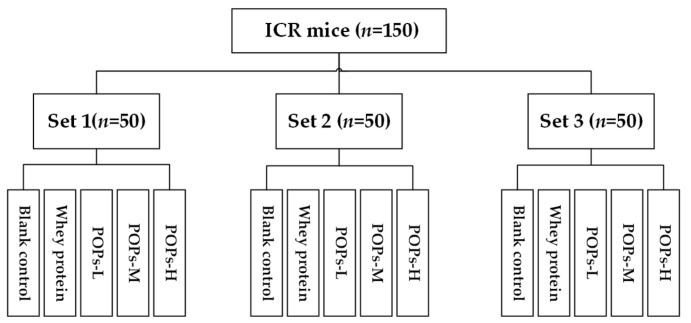
Grouping design of mice in the experiment.

As shown in Figure 1, after one week of acclimatization, the mice were randomly divided into three experimental sets (*n* = 50), namely, sets 1, 2, and 3. The mice in set 1 were subjected to a weight-loaded swimming test, the mice in set 2 were subjected to lactic acid and liver and muscle glycogen content examinations, and the mice in set 3 were used to test other mechanism-related indicators following 1.5 h of swimming without experiencing any load. Each set of mice was divided further into five intervention groups (*n* = 10): one blank control group, one whey protein group (0.50 g/kg·bw), and three POP intervention groups (POPs-L, POPs-M, POPs-H, and the intervention doses were 0.25, 0.50, and 1.00 g/kg·bw, respectively). The mice in the POP or whey protein groups received the POPs or whey protein solutions via oral administration, while distilled water was administered orally to mice in the blank control group. All groups were administered the selected samples by daily transoral gavage at 0.1 mL/10 g·bw for 30 days of continuous gavage intervention. The whey protein group was established to exclude the effect of protein intake alone on the anti-fatigue effect it experienced. Most of the doses administered in the previous studies concerning the anti-fatigue effects of bioactive peptides ranged from 0.11–1.0 g/kg·bw. Therefore, 0.5 g/kg·bw was selected as an intermediate dose in the study, and one high- and one low-dose group (1.0 and 0.25 g/kg·bw, respectively) were established to reflect the dose–effect relationship [5,6,7].

### 2.3. Weight-Loaded Swimming Test

After 30 days of gavage, mice from experimental set 1 were loaded with a 5% body weight of lead skin at the root of the tail and placed in a swimming tank (length × width × height: 50 cm × 50 cm × 40 cm, respectively) with a water depth of ≥30 cm and water temperature of 25 °C ± 1 °C, and the time from the beginning of the swimming test to the time of death caused by exhaustion was recorded as the time of the weighted swimming of the mice.

### 2.4. Blood Lactic Acid Determination

Following 30 days of gavage, mice from experimental set 2 were used to examine blood lactic acid (BLA) levels. Following the collection of 20 μL of blood from the tail tips of the mice, they were placed in 30°C water and swam without weight for 10 min. The collected blood samples were added to 0.48 mL of 1% sodium fluoride solution prior to swimming, 0 min after swimming, and then 20 min after swimming, and then thoroughly mixed until they turned transparent. Subsequently, the samples were mixed with 1.5 mL of protein precipitant and centrifuged at 4 °C and 3000 r/min for 10 min. The supernatant was then used to examine the blood lactate content for each group of mice by the colorimetric method of *p*-hydroxy biphenyl, according to the procedure presented in Table 1. A UV-2000 spectrophotometer (UNICO) was used to detect the absorbance value of each tube. The BLA concentration was calculated according to the Formula (1):

BLA concentration(g/L) = A/B, (1)

A was the absorbance value of test tube; B was the absorbance value of a standard tube.

The area under the BLA curve was calculated according to the Formula (2):

CS = 1/2 × (C0 + C1) × 10 + 1/2 × (C1 + C2) × 20, (2)

C0, C1, and C2 represent the BLA concentrations of the mice at baseline, 0, and 20 min after performing the swimming test, respectively. Cs represents the area under the BLA curve.

Protein precipitant: equal volumes of 10% sodium tungstate and 1/3 mol/L sulfuric acid, mixed with 28 times the volume of distilled water. Protein precipitant-NaF mixture: 1% NaF and 3 times the volume of precipitant mixture. Lactic acid standard application solution: 1.0 mL of lactic acid standard solution diluted and volume fixed to 100 mL. A total of 1.5% *p*-hydroxybiphenyl solution: 1.5 g of *p*-hydroxybiphenyl weighed and dissolved in 100 mL of hot 0.5% NaOH.

### 2.5. Determination of Liver and Muscle Glycogen Content

Mice from set 2 were sacrificed after one week of blood lactate content determination experiments under anesthesia, and their liver and muscle tissues were rapidly separated, rinsed with saline, blotted on filter paper, and assayed for their liver and muscle glycogen content according to the kit instructions.

### 2.6. Biochemical Assay

Mice in set 3 were forced to swim in 30 °C water without any loads for 1.5 h; then the mice were placed back into their cages. After resting for one hour, blood was obtained from the mice by removing the eyeball and then the liver; left-leg gastrocnemius and heart tissue samples were immediately dissected. The blood samples we obtained were placed in a refrigerator at 4 °C for 3 h and then centrifuged at 2000 r/min for 15 min after clotting, and the serum was separated. Serum LDH, CK, BUN, and blood glucose levels were measured using an Beckman AU480 chemistry analyzer (Beckman Coulter, Pasadena, CA, USA). The antioxidant indexes (SOD, GSH-PX activity, and MDA level) of cardiac muscle, liver, and gastrocnemius, and the activities of energy-metabolizing enzymes (PK, SDH, MDH, Na^+^-K^+^-ATPase, and Ca^2+^-Mg^2+^-ATPase) were measured according to the procedures specified by the kits, as presented in Section 2.1.

### 2.7. Quantitative Real-Time PCR

The expressions of nuclear respiratory factor 1 (NRF-1) and mitochondrial transcription factor A (mtTFA) in the gastrocnemius muscle of the mice in set 3 were examined by quantitative real-time PCR assay. 

Total RNA was extracted from the gastrocnemius muscle using a Trizol reagent (15596-018, Invitrogen, Carlsbad, CA, USA). RNA concentration and purity and integrity were tested, and then reverse transcription was performed by M-MLV reverse transcriptase (AM2044, Invitrogen, Carlsbad, CA, USA). The full-length sequence of the target gene mRNA was obtained from GenBank, and primer sequences were designed using Primer 5.0. Following a BLAST analysis, we obtained specific primer sequences. The primers and internal reference primers we used in the experiment are presented in Table 2. Real-time reverse-transcription PCR was performed using an ABI PRISM 7500 real-time PCR-detection system to detect the RNA expression of target genes with the specie primers. The cycling conditions were predenaturation at 95 °C for 2 min followed by 35 repeats of denaturation at 95 °C for 15 s and annealing for 30 s. Target mRNA values were determined by comparison to the control sample after being normalized to β-actin levels and calculated using the comparative cycle threshold (^ΔΔ^Ct) method.

### 2.8. Statistical Analysis

Statistical analyses were performed using SPSS software version 24 (SPSS Inc., Chicago, IL, USA). All values are presented as the mean ± standard deviation (SD). Differences between the groups were analyzed using the one-way analysis of variance (ANOVA) test with least significant difference (LSD) methods if the data were homogeneous. A value of *p* < 0.05 was considered statistically significant.

## 3. Results

### 3.1. Effect of POPs on the Body Weight of the Mice

**Figure 2:**
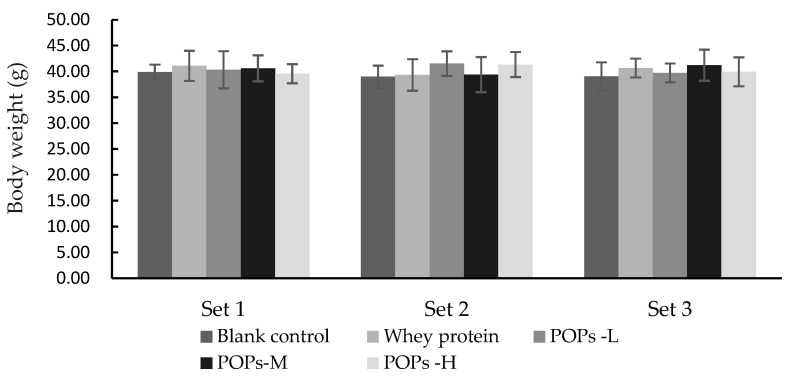
Effect of POPs on body weight of mice in each set. The data were analyzed for significance of difference by one-way analysis of variance (ANOVA) with the LSD test (*p* < 0.05); *n* = 10 per group. POPs-L, 0.25 g/kg·bw peanut oligopeptide group; POPs-M, 0.50 g/kg·bw peanut oligopeptide group; POPs-H, 1.00 g/kg·bw peanut oligopeptide group.

As shown in Figure 2, after 30 days of continuous gavage intervention, no significant differences were observed in the body weight values of the mice in each group (*p* > 0.05).

### 3.2. Effect of POPs on Exhaustive Swimming Time of Mice

**Figure 3:**
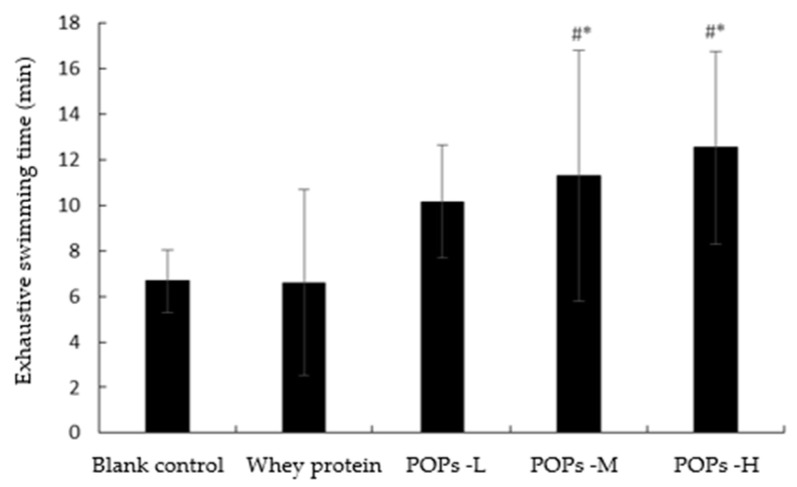
Effect of POPs on exhaustive swimming time of mice. The data were analyzed for significance of difference by one-way analysis of variance (ANOVA) with the LSD test; *n* = 10 per group. # represents significant difference compared with blank control; * represents significant difference compared with whey protein group. *p* < 0.05 indicates that the difference is significant. POPs-L, 0.25 g/kg·bw peanut oligopeptide group; POPs-M, 0.50 g/kg·bw peanut oligopeptide group; POPs-H, 1.00 g/kg·bw peanut oligopeptide group.

The weight-loaded swimming test reflected the decline in endurance and the progress of fatigue in the mice. As shown in Figure 3, The results show that compared with the blank control group, the mice in the medium- and high-dose POP groups presented significantly longer exhaustive swimming times (*p* < 0.05).

### 3.3. Effect of POPs on Blood Lactate Concentration Levels in Mice

**Figure 4:**
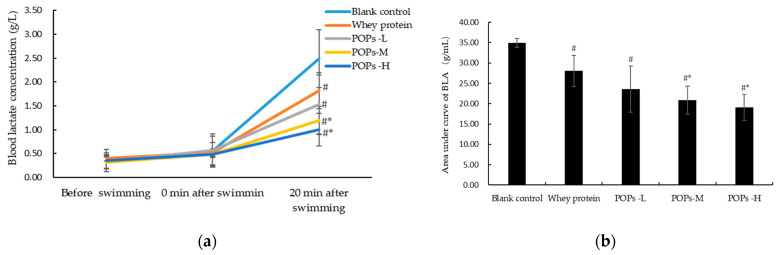
(**a**) Effect of POPs on blood lactate concentration in mice; (**b**) effect of POPs on area under curve of BLA in mice. Significance of main effects was analyzed by one-way analysis of variance (ANOVA) with the LSD test; *n* = 10 for each group. # *p* <0.05, versus blank control group; * *p* < 0.05, versus whey protein group. *p* < 0.05 indicates that the difference is significant. POPs-L, 0.25 g/kg·bw peanut oligopeptide group; POPs-M, 0.50 g/kg·bw peanut oligopeptide group; POPs-H, 1.00 g/kg·bw peanut oligopeptide group.

The change in blood lactic acid (BLA) concentration levels is an important index to evaluate the degree of fatigue progression that occurred in the mice. As shown in Figure 4a, there is no significant difference in BLA concentration levels prior to and at 0 min following the swimming test (*p* > 0.05). A total of 20 min following the swimming activity, the BLA concentration levels and area under the curve of BLA for the three POP groups were significantly lower than those in the blank control group (*p* < 0.05) and presented a dose–effect relationship (Figure 4a,b). In addition, compared with the whey protein group (Figure 4a,b), the BLA concentration level and the area under the curve of BLA in the POPs-M and POPs-H groups were also significantly reduced after 20 min of swimming (*p* < 0.05).

### 3.4. Effect of POPs on Serum Urea Nitrogen, Lactate Dehydrogenase, Creatine Kinase, and Blood Glucose Levels in Mice 

**Figure 5:**
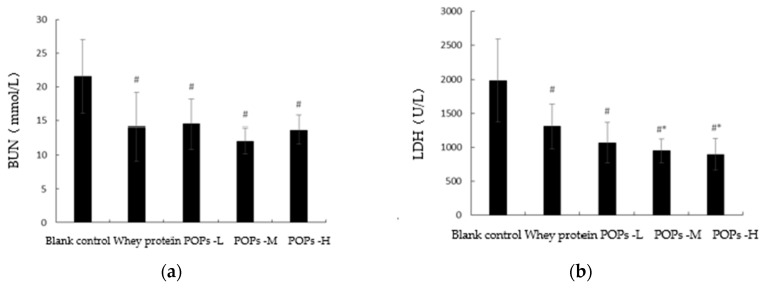

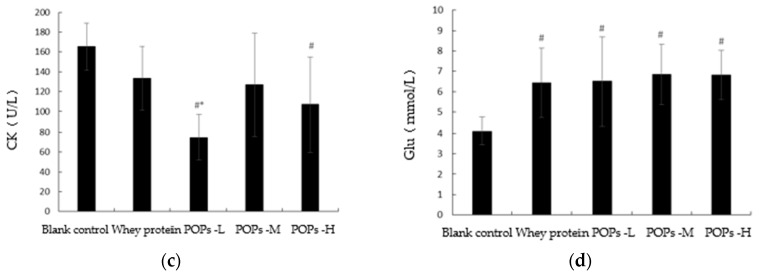
Effects of POPs on BUN (**a**), LDH (**b**), CK (**c**), Glu (**d**) levels in mice. Significance of main effects analyzed by one-way analysis of variance (ANOVA) with the LSD test; *n* = 10 for each group. # *p* <0.05, versus blank control group; * *p* < 0.05, versus whey protein group. *p* < 0.05 indicates that the difference is significant. LDH, lactate dehydrogenase; BUN, blood urea nitrogen; CK, creatine kinase; Glu, blood glucose. POPs-L, 0.25 g/kg·bw peanut oligopeptide group; POPs-M, 0.50 g/kg·bw peanut oligopeptide group; POPs-H, 1.00 g/kg·bw peanut oligopeptide group.

The changes occurring in serum urea nitrogen (BUN), lactate dehydrogenase (LDH), creatine kinase (CK), and blood glucose (Glu) levels can reflect the degree of fatigue progression of the organism experienced through different mechanisms. The results show that following 30 d of intervention, compared with the blank control group, the Glu levels of mice (Figure 5d) in the three POP groups were significantly increased (*p* < 0.05) and the levels of serum LDH (Figure 5b) and BUN (Figure 5a) were significantly decreased (*p* < 0.05), while the serum CK (Figure 5c) levels of mice in the POPs-L and POPs-H groups also presented a significant decrease (*p* < 0.05). Compared with the whey protein group, the serum LDH levels of mice (Figure 5b) in the POPs-M and POPs-H groups were significantly decreased (*p* < 0.05). 

### 3.5. Effect of POPs on the Content of Hepatic and Muscle Glycogen in Mice 

**Figure 6:**
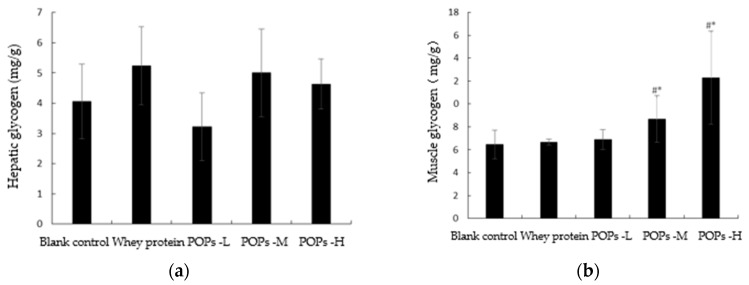
Effect of POPs on the content of hepatic (**a**) and muscle (**b**) glycogen in mice. The data were analyzed by one-way analysis of variance (ANOVA) with the LSD test; *n* = 10 for each group. # represents the significant difference compared with blank control group; * represents significant difference compared with whey protein group. *p* < 0.05 indicates that the difference is significant. POPs-L, 0.25 g/kg·bw peanut oligopeptide group; POPs-M, 0.50 g/kg·bw peanut oligopeptide group; POPs-H, 1.00 g/kg·bw peanut oligopeptide group.

Glycogen reserve is a key factor in the endurance levels of an organism during exercise. The results of our experiment show that, compared with the blank control and whey protein groups, the muscle glycogen content of mice (Figure 6b) in the POPs-M and POPs-H groups was significantly higher (*p* < 0.05); however, there was no significant difference evident between the hepatic glycogen levels of mice (Figure 6a) in each group (*p* > 0.05).

### 3.6. Effect of POPs on the Antioxidant Capacity of Mice

**Figure 7:**
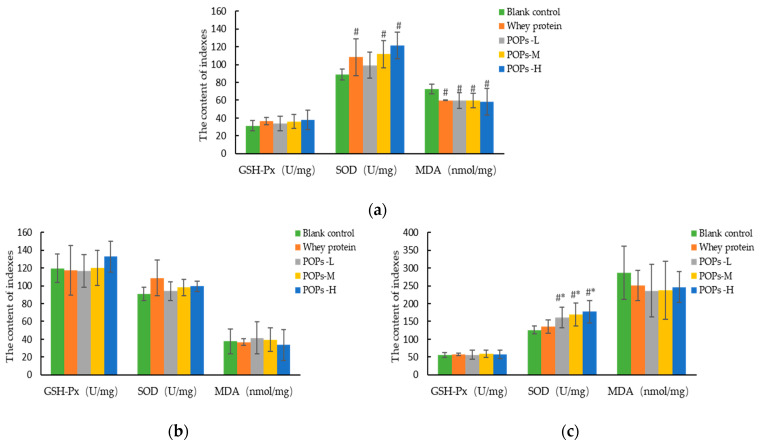
Effect of POPs on the antioxidant capacity of the cardiac muscle (**a**), liver (**b**), and gastrocnemius (**c**) in mice. The data were analyzed by one-way analysis of variance (ANOVA) with the LSD test; *n* = 10 for each group. # *p* <0.05, versus blank control group; * *p* < 0.05, versus whey protein group. *p* < 0.05 indicates that the difference is significant. SOD, superoxide dismutase; GSH-PX, glutathione peroxidase; MDA, malondialdehyde. POPs-L, 0.25 g/kg·bw peanut oligopeptide group; POPs-M, 0.50 g/kg·bw peanut oligopeptide group; POPs-H, 1.00 g/kg·bw peanut oligopeptide group.

Fatigue is closely related to oxidative stress, and the high number of free radicals produced by long-term or strenuous exercise is a key factor in the decline in muscle cell function. The results for the antioxidant capacity of the cardiac muscle show that, compared with the blank control group, malondialdehyde (MDA) content is significantly lower in the three POP groups (*p* < 0.05), and superoxide dismutase (SOD) activity is significantly higher in the POPs-M and POPs-H groups (*p* < 0.05) (Figure 7a). The results for the antioxidant capacity of gastrocnemius show that, compared with the blank control and whey protein groups, SOD activity is significantly higher in the three POP groups (*p* < 0.05) (Figure 7c). No significant difference was observed for the liver antioxidant capacity between the groups (*p* > 0.05) (Figure 7b).

### 3.7. Effect of POPs on the Activities of Pyruvate Kinase, Malate Dehydrogenase, and Succinate Dehydrogenase in Mice

**Figure 8:**
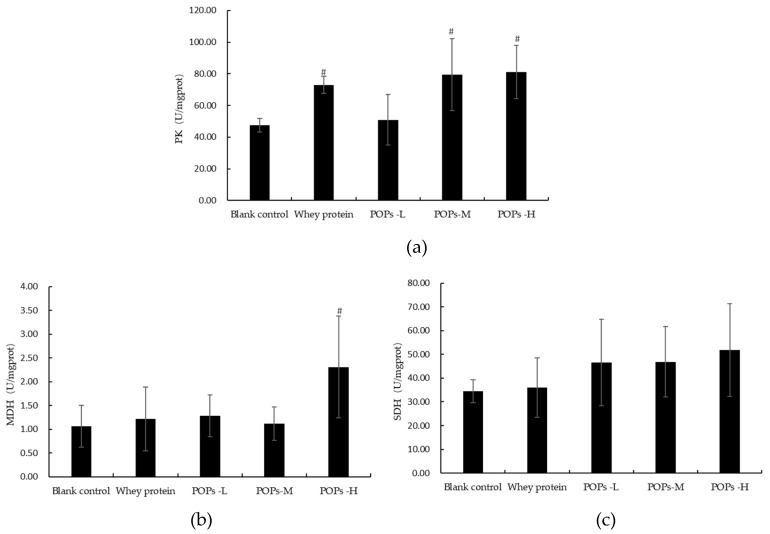
Effects of POPs on the activities of PK, MDA, and SDH in the skeletal muscles of mice. (**a**) Pyruvate kinase (PK); (**b**) malate dehydrogenase (MDH); and (**c**) succinate dehydrogenase (SDH). The data were analyzed by one-way analysis of variance (ANOVA) with the LSD test; *n* = 10 for each group. # represents significant difference compared with blank control group. *p* < 0.05 indicates that the difference is significant. POPs-L, 0.25 g/kg·bw peanut oligopeptide group; POPs-M, 0.50 g/kg·bw peanut oligopeptide group; POPs-H, 1.00 g/kg·bw peanut oligopeptide group.

Pyruvate kinase (PK), malate dehydrogenase (MDH), and succinate dehydrogenase (SDH) are key rate-limiting enzymes in the processes of aerobic oxidation and glycolysis, which are essential for catalyzing the synthesis of ATP. The results of our experiment show that the activity of PK is significantly higher in the POPs-M and POPs-H groups compared to the blank control group (Figure 8a), and the activity of MDH is significantly higher in the POPs-H group (Figure 8b) (*p* < 0.05). No significant difference can be observed for SDH activity occurring between the groups (*p* > 0.05) (Figure 8c).

### 3.8. Effect of POPs on the Activities of Na^+^-K^+^-ATPase and Ca^2+^-Mg^2+^-ATPase in Mice

**Figure 9:**
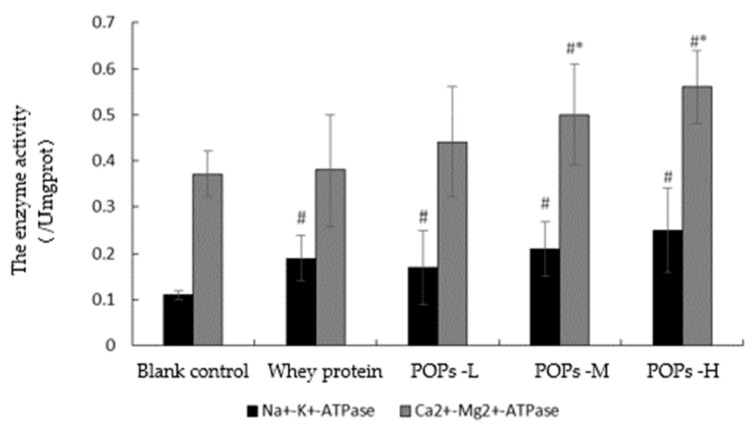
Effect of POPs on the activities of Na+-K+-ATPase and Ca^2+^-Mg^2+^-ATPase in mice. Significance of main effects analyzed by one-way analysis of variance (ANOVA) with the LSD test (*p* < 0.05); *n* = 10 for each group. # *p* <0.05, versus blank control group; * *p* < 0.05, versus whey protein group. Pops-L, 0.25 g/kg·bw peanut oligopeptide group; POPs-M, 0.50 g/kg·bw peanut oligopeptide group; POPs-H, 1.00 g/kg·bw peanut oligopeptide group.

Na^+^-K^+^-ATPase and Ca^2+^-Mg^2+^-ATPase are key enzymes in the process of ATP production. Prolonged or vigorous exercise causes a decrease in the activity of energy-metabolizing enzymes and reduces ATP synthesis, resulting in fatigue. As shown in Figure 9, our results show that compared with the blank control group, the activity of Na^+^-K^+^-ATPase in the three POP groups and the activity of Ca^2+^-Mg^2+^-ATPase in the POPs-M and POPs-H groups is significantly higher (*p* < 0.05). The activity of Ca^2+^-Mg^2+^-ATPase is significantly higher in the POPs-M and POPs-H groups compared with the whey protein group (*p* < 0.05).

### 3.9. Effect of POPs on the Mitochondrial Function of Gastrocnemius Muscles in Mice

**Figure 10:**
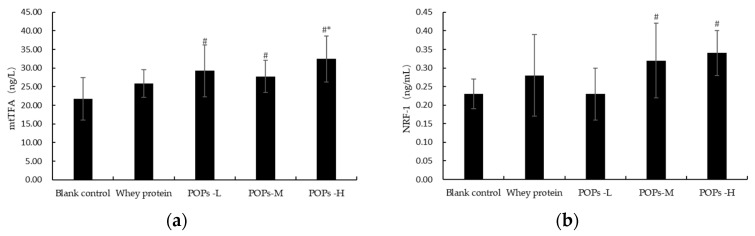
Effect of POPs on RNA expressions of mtTFA (**a**) and NRF-1 (**b**) in the gastrocnemius of mice determined by real-time PCR analysis. β-actin mRNA levels are used as a control. Significance of main effects analyzed by one-way analysis of variance (ANOVA) with the LSD test (*p* < 0.05); *n* = 6 for each group. # represents significant difference compared with blank control group; * represents significant difference compared with whey protein group. NRF-1, nuclear respiratory factor 1; TFAM, mitochondrial transcription factor A. POPs-L, 0.25 g/kg·bw peanut oligopeptide group; POPs-M, 0.50 g/kg·bw peanut oligopeptide group; POPs-H, 1.00 g/kg·bw peanut oligopeptide group.

Mitochondria are key sites for the intracellular synthesis of ATP, and mitochondrial transcription factor A (mtTFA) and nuclear respiratory factor 1 (NRF-1) are key factors in the process of mitochondrial biogenesis. Our results show that, when compared with the blank control group, mtTFA expression in the gastrocnemius muscles of the tested mice is significantly increased in the three POP groups, and the expression of NRF-1 is significantly increased in mice in the POPs-M and POPs-H groups (*p* < 0.05) (Figure 10). mtTFA expression is also significantly greater in the POPs-H group when compared with the whey protein group (*p* < 0.05) (Figure 10a).

## 4. Discussion

Strenuous exercise or physical activity can lead to a decrease in muscle function and endurance when performing exercise. A weight-loaded swimming test is one of the gold standards used in the research for evaluating the anti-fatigue effect, and the time of exhaustive swimming for mice can reflect their fatigue status and exercise endurance levels [22]. Our experimental results show that the intervention of POPs at both medium and high doses can significantly prolong the time of exhaustive swimming in mice, improve their exercise endurance levels, and produce anti-fatigue effects.

Carbohydrates are the main source of energy in the energy metabolism process and are mainly stored in the form of glycogen in more active metabolic sites, such as the muscles and liver, which can be broken down rapidly during exercise to supply energy, maintain blood glucose levels, and delay fatigue [23]. In this study, the POPs-intervention strategy significantly increased muscle glycogen and blood glucose levels in mice but did not display a significant dose–effect relationship, suggesting that POPs can delay the occurrence of fatigue by increasing muscle glycogen reserve levels, maintaining blood glucose levels and improving muscle function. In addition, we investigated the relationship between the anti-fatigue effect of POPs and the energy metabolic rate in muscle cells further by detecting the activity of key enzymes participating in the energy metabolic process. Pyruvate kinase (PK), malate dehydrogenase (MDH), and succinate dehydrogenase (SDH) are key rate-limiting enzymes that catalyze ATP synthesis and play an important role in information transfer and energy conversion processes during the tricarboxylic acid cycle, which can cause a decrease in ATP synthesis and lead to reduced muscle function [24]. Na^+^-K^+^-ATPase and Ca^2+^-Mg^2+^-ATPase play a role in maintaining intra- and extracellular osmotic pressure and regulating muscle contraction and diastole during energy metabolism [25]. The results show that POP intervention significantly increases the activities of PK, MDH, Na^+^-K^+^-ATPase, and Ca^2+^-Mg^2+^-ATPase in the gastrocnemius muscles of mice, indicating that POPs can delay the occurrence of fatigue by increasing the activity of key enzymes during energy metabolism, promoting the synthesis of ATP and regulating muscle contractions.

Vigorous exercise is often accompanied by an insufficient oxygen supply in the body, which, in turn, creates enhanced glycolytic pathways and lactic acid accumulation, leading to reduced muscle function and inducing soreness. Proteins and amino acids are degraded during exercise to produce high quantities of free ammonia, which inhibits the activity of energy-metabolizing enzymes and the synthesis of ATP [26]. Blood lactate acid (BLA) concentration and blood urea nitrogen (BUN) levels can reflect the accumulation status of metabolic substances in the body [5,7]. The results show that POP intervention can significantly delay the increase in BLA after swimming and reduce the area under the curve of BLA, showing a certain dose–effect relationship, and the effect produced was better than that of the whey protein. Meanwhile, the POP intervention also significantly reduced the level of serum BUN in mice, suggesting that POPs can produce an anti-fatigue effect by removing the accumulation of metabolic substances in the body.

Oxidative stress is a key fatigue-inducing factor. The high quantity of free radicals produced during exercise can damage the function of cells and mitochondria through lipid peroxidation and inhibit the synthesis of ATP, and the antioxidant capacity and mitochondrial function are important indicators reflecting the body’s ability to resist fatigue [27]. Superoxide dismutase (SOD) and glutathione peroxidase (GSH-Px) are critical components of the endogenous antioxidant defense mechanism, which have the function of scavenging free radicals, chelating metal ions, and mitigating oxidative damage [28,29,30]. Malondialdehyde (MDA) is the key factor that causes damage to the biofilm system during lipid peroxidation and is an important indicator for evaluating oxidative stress damage [29,30,31]. The experimental results show that the POP intervention significantly increases the activity of SOD in the myocardium and gastrocnemius muscle and decreases the level of myocardial MDA available, indicating that the POPs can exert anti-fatigue effects by improving the antioxidant capacity and reducing oxidative stress damage in the body. Damage to the cell membrane structure by free radicals leads to the escape of lactate dehydrogenase (LDH) and creatine kinase (CK) [32,33]. CK is mainly found in the cytoplasm and mitochondria, and it is directly related to muscle contraction and ATP regeneration processes [33]. LDH is one of the most important anaerobic enzymes for gluconeogenesis, which can catalyze the lactic acid degradation process [34]. The escape of CK inhibits energy metabolism and the escape of LDH causes lactic acid accumulation and aggravates the progression of fatigue [34]. In this study, the POP intervention significantly reduced the levels of serum LDH and CK in mice, and the effect was better than that of the whey protein, suggesting that the POPs can effectively reduce the damage of free radicals to cell membranes. In addition, free radicals also cause damage to subcellular structures. Mitochondria are key sites for energy metabolism, and mitochondrial dysfunction can severely affect muscle and nervous system functions and induce fatigue [35,36]. Mitochondrial transcription factor A (mtTFA) and nuclear respiratory factor 1 (NRF-1) are essential for mitochondrial gene expression and function; mtTFA promotes mitochondrial DNA replication and transcription processes and is regulated by NRF-1 [37,38]. The results of this experiment show that the POP intervention significantly increases expressions of mtTFA and NRF-1 in gastrocnemius mitochondria, suggesting that POPs improve mitochondrial function and encourage energy metabolism by reducing the damage of oxidative stress to mitochondrial membranes, thereby producing an anti-fatigue effect.

POPs are rich in essential amino acids, such as lysine, aspartic acid, glutamic acid, and leucine [20]. Aspartic and glutamic acids can chelate metal ions to inhibit the effect of oxidative stress. Leucine plays an important role in the synthesis of skeletal muscle proteins. Lysine can neutralize free-radical electrons generated during the oxidation of unsaturated fatty acids and is an important raw material for nerve cells and hemoglobin synthesis, which can effectively improve the body’s hypoxic and central fatigue states [21]. Whey protein is mainly composed of lactalbumins and lactoglobulins, which have various physiological activities. An analysis of the amino acid compositions of albumin and globulin revealed that albumin contains a high number of charged amino acid residues, including lysine (~5–10%), aspartic acid (~8–12%), and glutamic acid (~8–12%), a result fairly similar between whey protein and POPs that have important physiological functions and are important nutrients for living organisms [39,40]. This similarity may explain the comparable differences we observed for the majority of the blood parameters and few selective enzyme activities for whey protein and POP groups when compared to the blank control group. Moreover, POPs have a small molecular weight and can participate in energy metabolism and protein synthesis processes, providing a source of nitrogen for the body; peanut peptides can be absorbed directly by the intestinal mucosa, and their transit rate and absorption efficiency are better than protein and amino acids. Therefore, nutritional supplementation may also be one of the mechanisms through which POPs can alleviate and work against exercise-induced fatigue symptoms, and POP-M/POP-H exerted better effects than the whey protein.

In future studies, considering the limitations presented by in vivo studies, the specific anti-fatigue mechanisms of POPs can be investigated further using alternative experiments. Following our review of the relevant literature, we determined that a mouse C_2_C_12_ myogenic cell can be used to perform cellular experiments, which functions in a manner similar to human-derived myogenic cells. By using POPs to intervene in C_2_C_12_ myofibroblasts, we could successfully investigate their effect on myotubular cell activity, myotubular diameter, myotubular cell mitochondrial membrane potential, ATP concentration, and oxidative stress-related indexes, and we used transcriptomics to explore and identify potential mechanistic pathways and then validated them by qRT-PCR and Western blotting. Moreover, the ongoing research continues to probe the exact molecular mechanism by which POPs function against fatigue and explore the biological effect and optimal dose of POPs to produce anti-fatigue effects in humans.

## 5. Conclusions

In this study, the anti-fatigue effect and mechanisms of POPs were investigated by the continuous gavage of peanut peptides for 30 days in ICR mice. We suggest that POPs had the effect of improving the anti-fatigue effect in mice through the mechanisms of promoting better energy metabolism, clearing the accumulation of metabolic substances in the body, improving the body’s antioxidant capacity, and reducing the damage of oxidative stress to cellular and mitochondrial functions.

## Figures and Tables

**Table 1:** Procedure used for the determination of blood lactate content by a colorimetric assay with *p*-hydroxybiphenyl.

Reagent	Blank Control Tube (mL)	Standard Control Tube (mL)	Test Tube (mL)
Protein precipitant–NaF mixture	0.5		
Lactic acid standard-application solution		0.5	
Supernatant solution			0.5
4%CuSO_4_	0.1	0.1	0.1
Concentrated sulfuric acid	3	3	3
Placed in boiling water for 5 min after being mixed; then placed in cold water for 10 min			
1.5% *p*-hydroxybiphenyl solution	0.1	0.1	0.1

**Table 2:** Primer information for the experiment.

Objective Gene	Primer	Sequences	Length	Temperature (°C)
β-actin	Forward	GATTACTGCTCTGGCTCCTAG	147 bp	62
	Reverse	GACTCATCGTACTCCTGCTTGC		
NRF-1	Forward	TATGGCGGAAGTAATGAAAGACG	101 bp	60
	Reverse	CAACGTAAGCTCTGCCTTGTT		
mtTFA	Forward	AGGTCCAGCTCACTAACTGC	217 bp	62
	Reverse	TGTATGCTGTGGTTTCCCAGT		

## Data Availability

The data presented in this study are available on request from the corresponding author. The data are not publicly available due to privacy. The studies not involving humans.
